# Metagenomic characterization of the porcine respiratory virome on farms in northern Kazakhstan

**DOI:** 10.3389/fvets.2026.1926297

**Published:** 2026-09-07

**Authors:** Nurbol Saktaganov, Nuray Ongarbayeva, Nailya Klivleyeva, Tatyana Glebova, Assem Baimukhametova, Mereke Kalkozhayeva, Astan Aliyev, Andrey Komissarov, Nikita Yolshin, Artem Fadeev, Daria Danilenko, Primkul Ibragimov, Kobey Karamendin

**Affiliations:** 1 Laboratory of Biochemistry of Viruses, Department of Virology, Research and Production Center for Microbiology and Virology, Almaty, Kazakhstan; 2 Laboratory of Molecular Virology, Smorodintsev Research Institute of Influenza, Saint Petersburg, Russia; 3 Department of Biological Safety, University Board, Kazakh National Agrarian Research University, Almaty, Kazakhstan

**Keywords:** Kazakhstan, metagenome, porcine virome, sequencing, surveillance

## Abstract

Studies of the porcine respiratory virome are essential for understanding the complex viral communities in the porcine respiratory tract and their roles in health and disease. Characterizing the respiratory virome provides insights into the diversity, prevalence, and dynamics of pathogenic and commensal viruses, thereby facilitating the identification of emerging and previously unrecognized respiratory pathogens. Metagenomic next-generation sequencing (mNGS) is a method for analyzing viral communities, enabling comprehensive monitoring of circulating and co-detected pathogens in animals. In this study, mNGS on the Illumina platform was used to characterize the porcine respiratory virome in Kazakhstan. The study included 150 nasopharyngeal swabs collected from commercial pig farms in northern Kazakhstan. Viral sequences were assigned to the families *Parvoviridae, Herpesviridae, Picornaviridae, Astroviridae*, and *Anelloviridae*. High coverage was achieved for Porcine cytomegalovirus, Porcine parvovirus 5, and Porcine parvovirus 7, including reconstruction of a nearly complete PPV7 genome. Significant variability in mapped read counts and sequencing depth was observed across samples, reflecting heterogeneity in the detection of viral nucleic acids within the sampled population. Phylogenetic analysis showed that all identified viruses belong to globally circulating evolutionary lineages and do not form new divergent clusters. Moreover, respiratory and gastrointestinal virus sequences were often detected simultaneously in nasopharyngeal samples, indicating a complex composition of viral nucleic acids in the upper respiratory tract of pigs. Pigs are important reservoirs and intermediate hosts for zoonotic viruses. Metagenomic studies contribute not only to animal health and disease control but also to One Health initiatives by strengthening early warning systems for pathogens with epidemic or pandemic potential. The results demonstrate the high viral diversity in domestic pigs in Kazakhstan and confirm the effectiveness of mNGS as a tool for epizootiological surveillance and molecular monitoring of viral infections in livestock.

## Introduction

Pigs are important farm animals, playing a key role in food security in some regions worldwide, and can serve as reservoirs for a wide range of viral pathogens. Of particular zoonotic concern are influenza viruses A, C, and D (family *Orthomyxoviridae*), which can infect humans, birds, and other animals (1–3). In addition to influenza, coinfections with the porcine reproductive and respiratory syndrome virus (PRRSV), porcine astroviruses (PoAstV4), porcine circoviruses (PCV2-PCV3), and parainfluenza viruses (PPIV-1 and PPIV-2) cause significant economic losses in the pig industry. The multifactorial nature of these diseases leads to high mortality in young animals and reduced productivity (4, 5). Risk analysis and historical data on zoonotic events highlight pigs' potential as a source of interspecies virus transmission (6).

Classical diagnostic methods (such as PCR and serological tests) effectively detect known pathogens but are limited in identifying new or poorly characterized viruses because they rely on predetermined targets (7, 8). Porcine respiratory diseases are a leading cause of economic losses in industrial pig farming, resulting in reduced productivity, increased mortality among young animals, and significant veterinary costs (9–11). In this context, metagenomic approach using next-generation sequencing (NGS) technologies is becoming an important tool for studying the viral composition of clinical samples, enabling targetless identification of pathogens without prior knowledge of their genomic sequences (12–15). For example, a metagenomic study of Tibetan pigs with respiratory disease symptoms identified 18 viruses from 15 families, including Porcine circovirus-2 (PCV2) and several previously unknown viral strains (16). A similar approach was applied to tissues from sick pigs, where respiratory and multisymptomatic viruses, including *Astroviridae* and *Arteriviridae*, were detected (17).

The results of these studies demonstrate the high value of metagenomic methods in veterinary virology and their importance for the comprehensive analysis of porcine respiratory diseases. In Kazakhstan, prior monitoring of influenza A virus strains in domestic pigs has reported low-level but measurable detection of swine influenza virus nucleic acid. This highlights the need for ongoing molecular surveillance and expanded research using NGS approaches (18–20).

Despite the growing body of data on porcine viruses generated by high-throughput sequencing technologies, knowledge of viral diversity in pigs in Kazakhstan remains limited. According to the National Statistics Bureau of the Republic of Kazakhstan, as of 2025, the country's total swine population was 475,000, distributed unevenly across regions. The highest concentrations of animals were recorded in the North Kazakhstan (132,700), Karaganda (78,500), and Pavlodar (82,500) regions, due to the highly developed infrastructure for intensive industrial pig farming. Significant populations were also recorded in the Kostanay (67,200) and Akmola (32,400) regions. In other regions of the country, pig populations were significantly lower, ranging from 200 (Atyrau region) to 23,100 (Almaty region).

Most studies in the region have focused on individual pathogens, while a comprehensive characterization of the porcine respiratory virome has not been conducted. Therefore, this study aimed to investigate the viral diversity of the swine respiratory tract in Kazakhstan using Illumina metagenomic sequencing and to reconstruct and genetically characterize the identified viral genomes. The study aimed to establish a baseline of virome composition and diversity under normal (non-outbreak) conditions, serving as a foundation for future comparative studies with diseased cohorts, consistent with a surveillance rather than a diagnostic design.

## Materials and methods

### Samples collection

**Figure 1:**
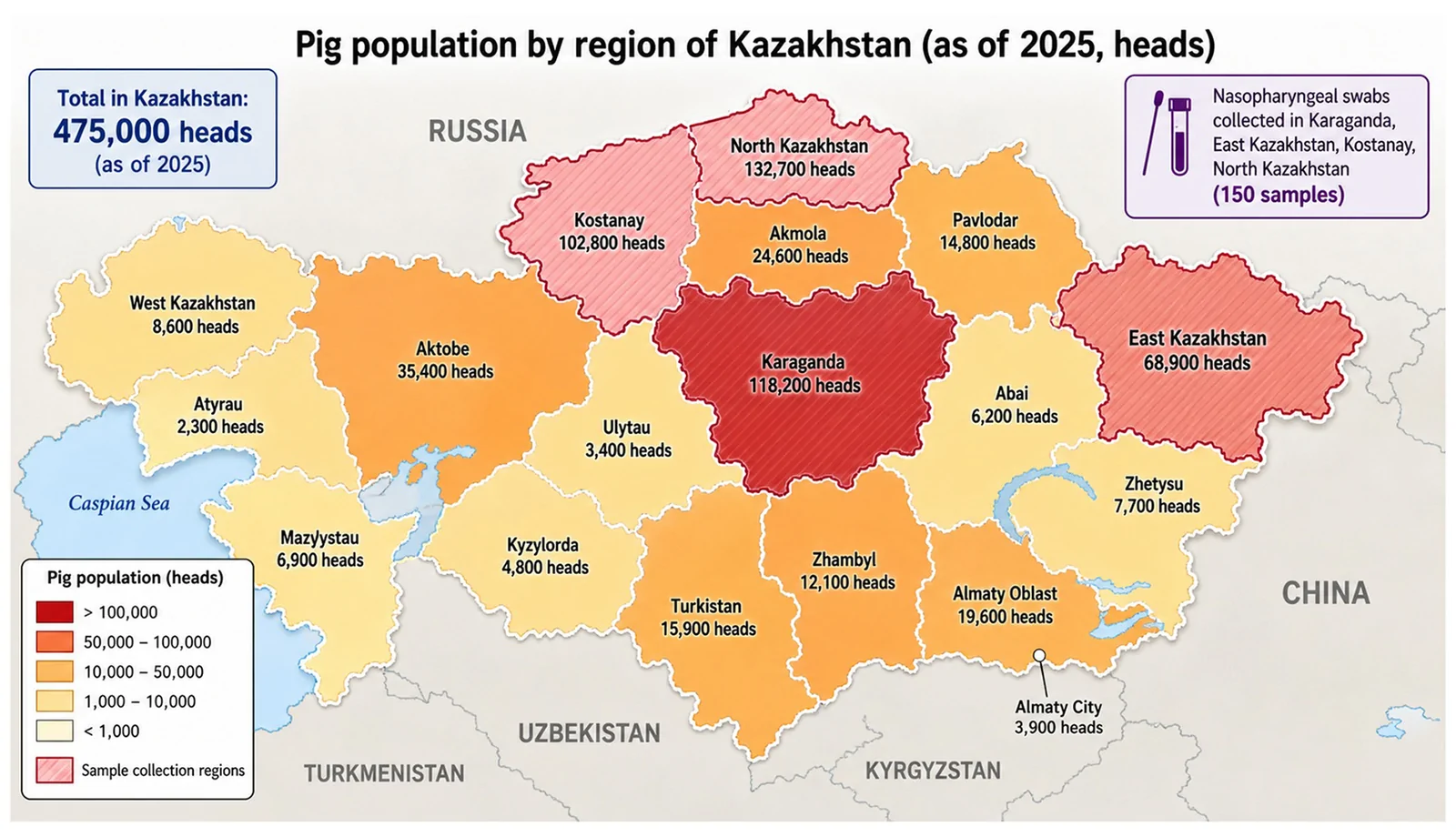
Distribution of swine population by regions in Kazakhstan (2025) and the geographical location of areas covered by the epidemiological survey.

Nasopharyngeal swabs were collected from pigs in the Karaganda, East Kazakhstan, Kostanay, and North Kazakhstan regions (Figure 1) during 2024–2025, where intensive pig farming is common. Swabs were collected as part of routine herd health monitoring of asymptomatic animals. Samples were placed in transport medium (VTM199) containing antibiotics (penicillin−2,000 U/ml, streptomycin−2 mg/ml, gentamicin−50 μg/ml, nystatin−50 U/ml) and bovine serum albumin at a final concentration of 0.5%, then transported in liquid nitrogen (−196 °C) in a Dewar vessel until laboratory processing, preserving viral nucleic acids. Prior to laboratory processing, samples were stored in a low-temperature freezer at -70 °C.

### Samples processing and double-stranded cDNA synthesis

Nasopharyngeal swabs in viral transport medium were centrifuged at 3,200 rpm for 15 min in an Eppendorf 5417R centrifuge (FA-45-24-11 rotor; Eppendorf, Germany). The supernatant was filtered through 0.22 μm membrane filters (Membrane Solutions, USA). To remove free host and microbial nucleic acids, the filtrate was treated with a nuclease mixture containing Benzonase^®^ (Sigma-Aldrich, USA), TURBO DNase™, DNase I, RNase A, and RNase T1 (Thermo Fisher Scientific, Lithuania). Viral nucleic acids were extracted using the QIAamp Viral RNA Mini Kit (Qiagen, Germany) following the manufacturer's instructions.

The SMART-9N protocol, with template-switching technology, was used for cDNA synthesis. One microliter of SMART-9N primer (NEB bRT-9N: 5′-AAGCAGTGGTATCAACGCAGAGTACNNNNNNNNN-3′; 2 μM) and one microliter of dNTP mixture (10 mM of each nucleotide) were added to 10 μl of isolated RNA. The mixture was incubated at 65 °C for 5 min and then immediately cooled on ice. Then, 4 μl of First-Strand Buffer, 1 μl of 0.1 M DTT, 1 μl of RNaseOUT™ RNase inhibitor, 1 μl of SSP template-switching oligonucleotide (5′-GCTAATCATTGCAAGCAGTGGTATCAACGCAGAGTACATrGrGrG-3′; 2 μM), and 1 μl of SuperScript IV Reverse Transcriptase enzyme (Thermo Fisher Scientific, USA) were added. The reverse transcription reaction was carried out at 42 °C for 90 min, followed by enzyme inactivation at 70 °C for 10 min. The resulting double-stranded cDNA was amplified using Q5 High-Fidelity DNA Polymerase (New England Biolabs, USA) and the NEB PCR universal primer (5′-AAGCAGTGGTATCAACGCAGAGT-3′). Amplification was performed under the following conditions: initial denaturation at 98 °C for 45 s; 30 cycles including denaturation at 98 °C for 15 s, annealing at 62 °C for 15 s, and elongation at 65 °C for 5 min; final elongation at 65 °C for 10 min (21). PCR products were purified using AMPure XP magnetic beads (Beckman Coulter, UK) at a 1:1 ratio and quantified using the Qubit dsDNA High Sensitivity Assay Kit on a Qubit 3.0 fluorometer (Thermo Fisher Scientific, USA). The resulting double-stranded cDNAs were used for library preparation. It is known that SMART-9N facilitates metagenomic sequencing of both DNA and RNA viruses (22), thereby enabling the recovery of DNA virus sequences.

### Library preparation, high throughput sequencing and bioinformatics

For high-throughput sequencing, 150 DNA libraries, each corresponding to one porcine nasopharyngeal sample, were prepared using the Illumina DNA Prep Kit (Illumina, USA) according to the manufacturer's protocol. Quality control and fragment distribution assessment were performed on an Agilent 2,100 Bioanalyzer (Agilent Technologies, USA). Sequencing was performed on the Illumina NextSeq 2,000 platform with the NextSeq 1,000/2,000 P2 Reagents (100 Cycles) v3 kit (Illumina) at the A.A. Smorodintsev Research Institute of Influenza (St. Petersburg, Russia).

Obtained reads were quality-assessed using FastQC v0.12.1 and preprocessed with fastp to remove adapter sequences and low-quality reads (23). Taxonomic classification of reads was performed using Kraken2 v2.1.7.1 (24) with the core_nt database. Samples in which at least 100 reads were classified as viral were selected for further analysis. Reads assigned to the respective viral taxa were subjected to reference-based genome assembly; partial genomes were recovered from all selected samples, and complete genomes were obtained when coverage was sufficient. For each identified virus, the corresponding reference sequences were downloaded from the GenBank database. Read alignment to reference genomes was performed using the BWA (25) software package. Alignment processing and BAM file manipulation were performed using SAMtools v1.20 (26). Consensus sequences were generated using the iVar v1.4.3 (27) tool based on the resulting alignments. To confirm the taxonomic affiliation of viral sequences, BLASTn and BLASTx were used to compare the resulting sequences with reference records in the GenBank database. The resulting consensus sequences were used for subsequent analysis of viral diversity and assessment of the taxonomic composition of viruses circulating in the study farms of Kazakhstan.

### Phylogenetic analyses

Multiple nucleotide sequences were aligned using the MAFFT version 7 algorithm (28). Phylogenetic trees were constructed using the maximum-likelihood method in MEGA 12 (29), with the most appropriate nucleotide substitution model selected automatically. Topological reliability was assessed using the bootstrap method with 1,000 pseudoreplicates. Viral sequences were selected for phylogenetic analysis based on three criteria applied jointly: prioritizing maximum geographical representation of the target locus to enable robust comparison with reference strains from multiple regions, genome/gene coverage exceeding 70% where achievable, and adequate representation of the relevant genotype diversity within each viral species.

## Results

A total of 150 porcine nasopharyngeal samples were collected in swine farms of northern Kazakhstan. The sequencing run yielded more than 102 million reads, averaging about 680,000 per sample. Most samples had high sequencing depth, providing sufficient sensitivity to detect viral sequences and support subsequent analysis of viral diversity. A wide range of DNA- and RNA-containing viruses from the *Parvoviridae, Astroviridae, Herpesviridae, Picornaviridae*, and *Anelloviridae* families was detected, indicating the presence of diverse viral sequences on pig farms in Kazakhstan.

**Figure 2:**
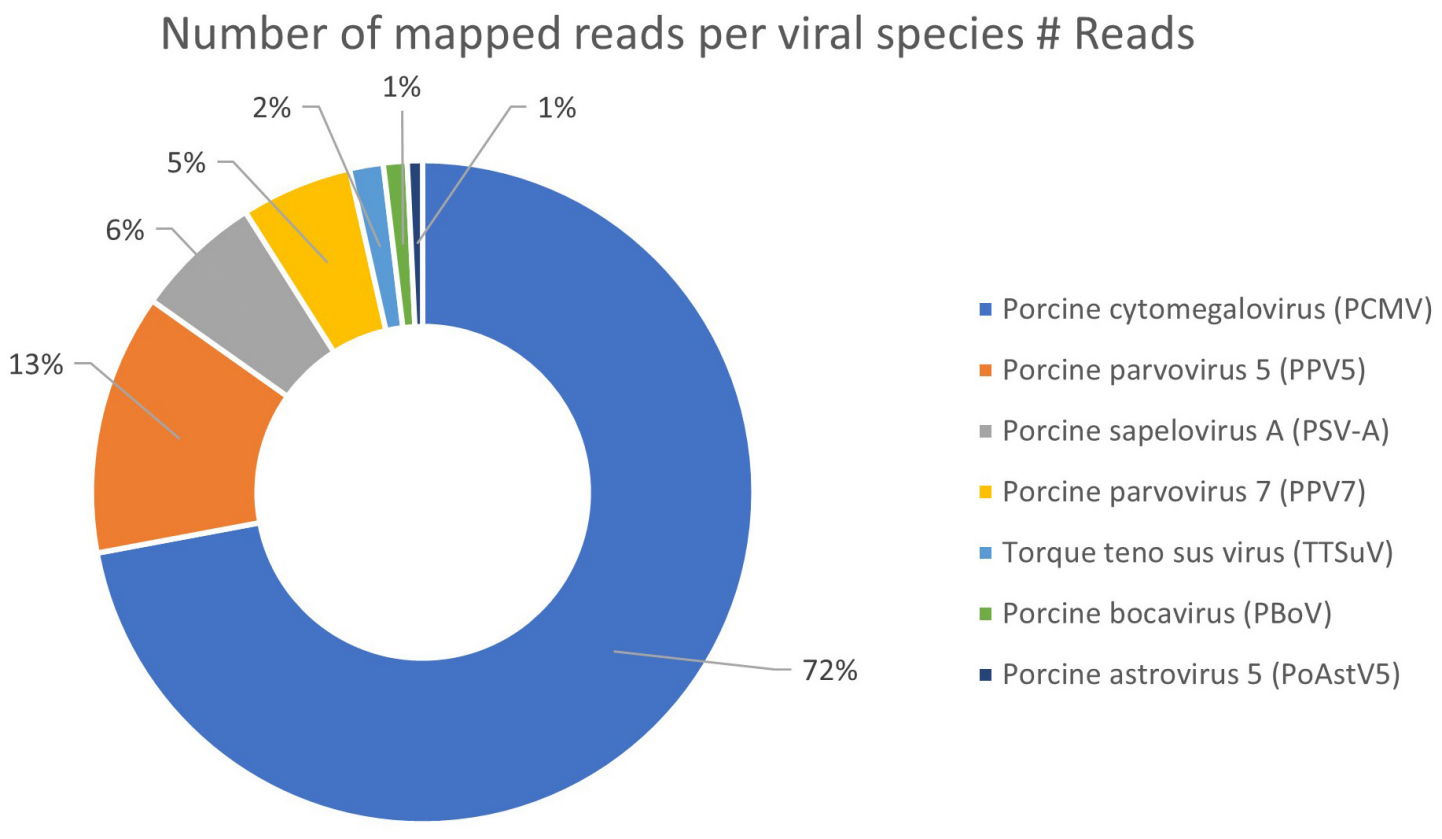
Number of mapped reads per viral species.

The highest number of mapped reads was observed for Porcine cytomegalovirus (PCMV)−95,484 reads in sample WD4885–with average and median coverage depths of 84.6 × and 12 ×, respectively, reflecting uneven read distribution across the herpesvirus genome (Figure 2). Porcine parvovirus 5 (PPV5) had the highest average coverage depth−361.2 × with 16,879 reads–indicating a highly complete genome reconstruction and robust alignment. For Porcine parvovirus 7 (PPV7), the mean and median coverage depths were 218.7 × and 107 ×, respectively, with 7,195 reads, indicating a nearly complete genome reconstruction. Porcine sapelovirus A (PSV-A) was represented by 8,236 reads with a mean coverage depth of 124.3 × and a median coverage of 49 ×, confirming its high abundance in sample WD4860. Porcine bocavirus (PBoV) showed moderate abundance (1,539 reads) with a mean coverage depth of 40.3 × and a median coverage of 4 ×, indicating a partial genome reconstruction. Porcine astrovirus 5 (PoAstV5) was characterized by a low read count (990) and limited depth of coverage (17.4 × ), consistent with a fragmented reconstruction of genomic regions. Torque teno sus virus (TTSuV) was detected in sample WD4891 (2,177 reads) with an average coverage depth of 96.8 × and a median coverage of 14 ×, reflecting its presence alongside significant heterogeneity in read distribution across the genome. The identified viral sequences showed high amino acid identity to GenBank reference sequences (87.87–100%), confirming the accuracy of the taxonomic classification and annotation (Table 1).

**Table 1:** Characteristics of viral sequences identified in porcine nasopharyngeal samples.

Family	Genus	Virus	Genome type	Sample	Protein name	Accession	Amino acid identity (%)	Query coverage (%)	E–value
Parvoviridae	Bocaparvovirus	Porcine bocavirus (PBoV)	ssDNA	WD4943	VP1 protein [Porcine bocavirus 1]	AHK27019	99.33	100	0.0
Astroviridae	Mamastrovirus	Porcine astrovirus 5 (PoAstV5)	+ssRNA	WD4928	ORF1ab [Porcine astrovirus 5]	UHY36853	94.14	63	0.0
Herpesviridae	Roseolovirus.	Porcine cytomegalovirus (PCMV)	dsDNA	WD4885	DNA polymerase [Suid betaherpesvirus 2]	AAF80108	100	100	0.0
Parvoviridae	undetermined	Porcine parvovirus 5 (PPV5)	ssDNA	WD4860	capsid protein [Porcine parvovirus 5]	QBA84739	95.75	46	0.0
Parvoviridae	Chaphamaparvovirus	Porcine parvovirus 7 (PPV7)	ssDNA	WD4860	nonstructural protein 1 [Porcine parvovirus 7]	QBQ18679	89.88	50	0.0
Picornaviridae	Sapelovirus	Porcine sapelovirus A (PSV–A)	+ssRNA	WD4860	polyprotein [Porcine sapelovirus A (PSV–A)]	NP_653145	87.87	79	0.0
Anelloviridae	Iotatorquevirus/Kappatorquevirus	Torque teno sus virus (TTSuV)	Circular ssDNA	WD4891	ORF1, partial [Torque teno virus]	QBA84169	92.64	25	2e−112

To confirm the metagenomic classification results, a reference-based assembly of viral genomes was conducted. This approach enabled us to reconstruct genomic sequences of varying completeness for viruses in the *Parvoviridae, Herpesviridae, Astroviridae, Picornaviridae*, and *Anelloviridae* families. The key characteristics of the resulting assemblies are presented in Table 2.

**Table 2:** Viral genomes reconstructed using the reference–based assembly.

Sample	Virus species	% of genome	Genome	Mapped	Unmapped	Median_cov	Total_reads	Reference_ID
**WD4830**	Suid betaherpesvirus 2	19.66%	no	4,838	685,499	0	690,337	NC_022233.1
**WD4834**	Porcine parvovirus 7	49.81%	no	1,003	273,346	2	274,349	NC_040562.1
**WD4860** ^*^	Porcine parvovirus 5	57.43%	no	16,879	782,722	9	799,601	NC_023020.1
**WD4860** ^*^	Porcine parvovirus 7	98.92%	almost	7,195	792,406	107	799,601	NC_040562.1
**WD4860** ^*^	Porcine sapelovirus A (PSV–A)	73.38%	no	8,236	791,365	49	799,601	NC_003987.1
**WD4865**	Porcine parvovirus 5	25.77%	no	1,312	892,476	0	893,788	NC_023020.1
**WD4874**	Porcine parvovirus 5	35.50%	no	10,515	1,174,155	0	1,184,670	NC_023020.1
**WD4874**	Porcine parvovirus 7	62.12%	no	10,018	1,174,652	22	1,184,670	NC_040562.1
**WD4875**	Porcine parvovirus 5	40.31%	no	2,450	947,797	0	950,247	NC_023020.1
**WD4876**	Porcine parvovirus 5	23.88%	no	224	887,475	0	887,699	NC_023020.1
**WD4881**	Porcine parvovirus 5	22.74%	no	505	965,795	0	966,300	NC_023020.1
**WD4884**	Suid betaherpesvirus 2	82.81%	no	192,468	703,397	29	895,865	NC_022233.1
**WD4885** ^*^	Suid betaherpesvirus 2	76.71%	no	95,484	750,635	12	846,119	NC_022233.1
**WD4891** ^*^	Torque teno sus virus k2a	67.75%	no	2,177	943,626	14	945,803	NC_014092.2
**WD4892**	Suid betaherpesvirus 2	2.84%	no	364	585,494	0	585,858	NC_022233.1
**WD4897**	Suid betaherpesvirus 2	2.59%	no	366	736,855	0	737,221	NC_022233.1
**WD4918**	Porcine endogenous retrovirus E	2.13%	no	4,997	521,199	0.0	526,196	NC_003059.1
**WD4922**	Suid betaherpesvirus 2	1.47%	no	619	479,321	0	479,940	NC_022233.1
**WD4928** ^*^	Porcine astrovirus 5	77.42%	almost	990	575,201	14.0	576,191	NC_023636.1
**WD4937**	Porcine parvovirus 7	90.47%	almost	448	554,502	9	554,950	NC_040562.1
**WD4943** ^*^	Bocavirus pig/SX/China/2010	53.63%	no	1,539	409,050	4.0	410,589	NC_038537.1

^*^Taken for phylogenetic analyses.

The most complete genomic sequence of Porcine parvovirus 7 (PPV7) was reconstructed from sample WD4860, with 98.92% reference genome coverage and a median depth of 107 × . High coverage was also achieved for the second PPV7 isolate (90.47%), Porcine cytomegalovirus (up to 82.81%), Porcine astrovirus 5 (77.42%), and Porcine sapelovirus A (PSV-A) (73.38%). Partial genomic sequences suitable for further comparative and phylogenetic analysis were obtained for Porcine bocavirus, Torque teno sus virus (TTSuV), and Porcine parvovirus 5.

Viral coinfections were detected in several samples. The most pronounced coinfection occurred in sample WD4860, where Porcine parvovirus 5, Porcine parvovirus 7, and Porcine sapelovirus A (PSV-A) were detected simultaneously. Additionally, PPV5 and PPV7 were co-detected in samples WD4874 and WD4881. These data indicate the co-detection of multiple viral sequences within a single animal and confirm the utility of metagenomic sequencing for identifying potential mixed viral infections, though co-detection alone does not confirm concurrent active infection. To assess the prevalence of the identified viruses, detection rates were analyzed across nasopharyngeal samples from pigs (Table 3).

**Table 3:** Frequency of virus detection in nasopharyngeal samples of pigs according to metagenomic sequencing data.

Virus species	Positive samples	Frequency (%)
Porcine parvovirus 5 (PPV5)	6	4.0
Porcine parvovirus 7 (PPV7)	5	3.3
Porcine cytomegalovirus (PCMV)	5	3.3
Porcine astrovirus 5 (PoAstV−5)	1	0.7
Porcine sapelovirus A (PSV–A)	1	0.7
Torque teno sus virus (TTSuV)	1	0.7
Porcine bocavirus (PBoV)	1	0.7
Porcine endogenous retrovirus E (PERV–E)	1	0.7

Author Queries

Among the detected viruses, Porcine parvovirus 5 (PPV5) showed the highest detection rate, found in 6 of 150 samples (4.0%), while Porcine parvovirus 7 (PPV7) and Porcine cytomegalovirus (PCMV) were each detected in 5 samples (3.3%). Other viruses, including Porcine astrovirus 5, Porcine sapelovirus A, Torque teno sus virus, Porcine bocavirus, and Porcine endogenous retrovirus E, were detected sporadically at a rate of 0.7%. Overall, viral sequences were detected in 21 of 150 samples (12.0%), indicating a measurable detection rate of multiple viral agents in the sampled pig population and the presence of co-detected viral nucleic acids in some animals' nasopharyngeal samples. The relatively higher detection rates of parvoviruses and cytomegalovirus suggest these agents are more commonly present in the sampled porcine respiratory tract, though their functional role, if any, in shaping the local viral community cannot be established from detection data alone.

### Phylogenetic relationships of porcine respiratory viruses detected in Kazakhstan

**Figure 3:**
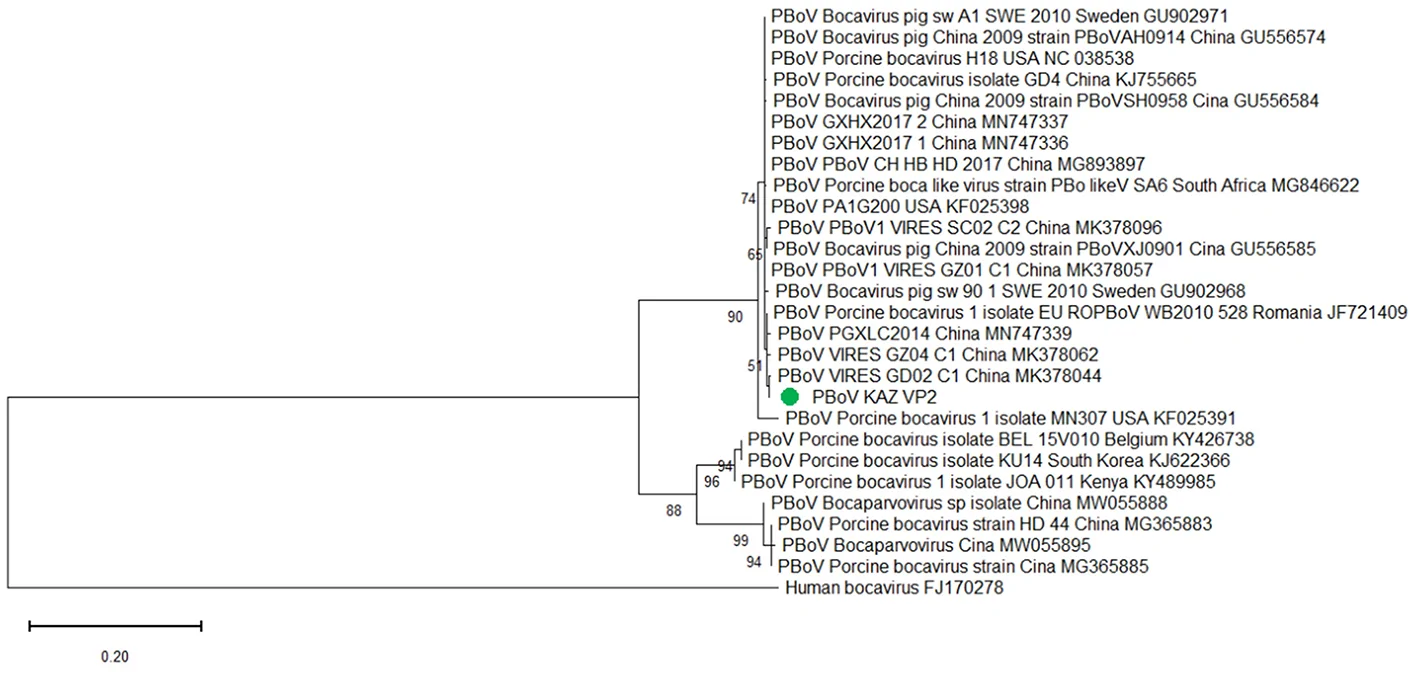
Phylogenetic analysis of Porcine bocavirus. The green circle indicates the Kazakhstan isolate (PBoV/KAZ/WD4943/2025).

For Porcine bocavirus, the VP1 gene sequence was used. This gene encodes the major structural protein of the capsid and important for phylogenetic analyses within a species. It was determined that the PBoV/KAZ/WD4943/VP2/2025 isolate identified in this study clusters within the Porcine bocavirus 1 (PBoV-1)-like group (Figure 3). The Kazakhstan sequence formed a common cluster with isolates previously described in China, the United States, and Europe, including strains from Romania and Sweden.

**Figure 4:**
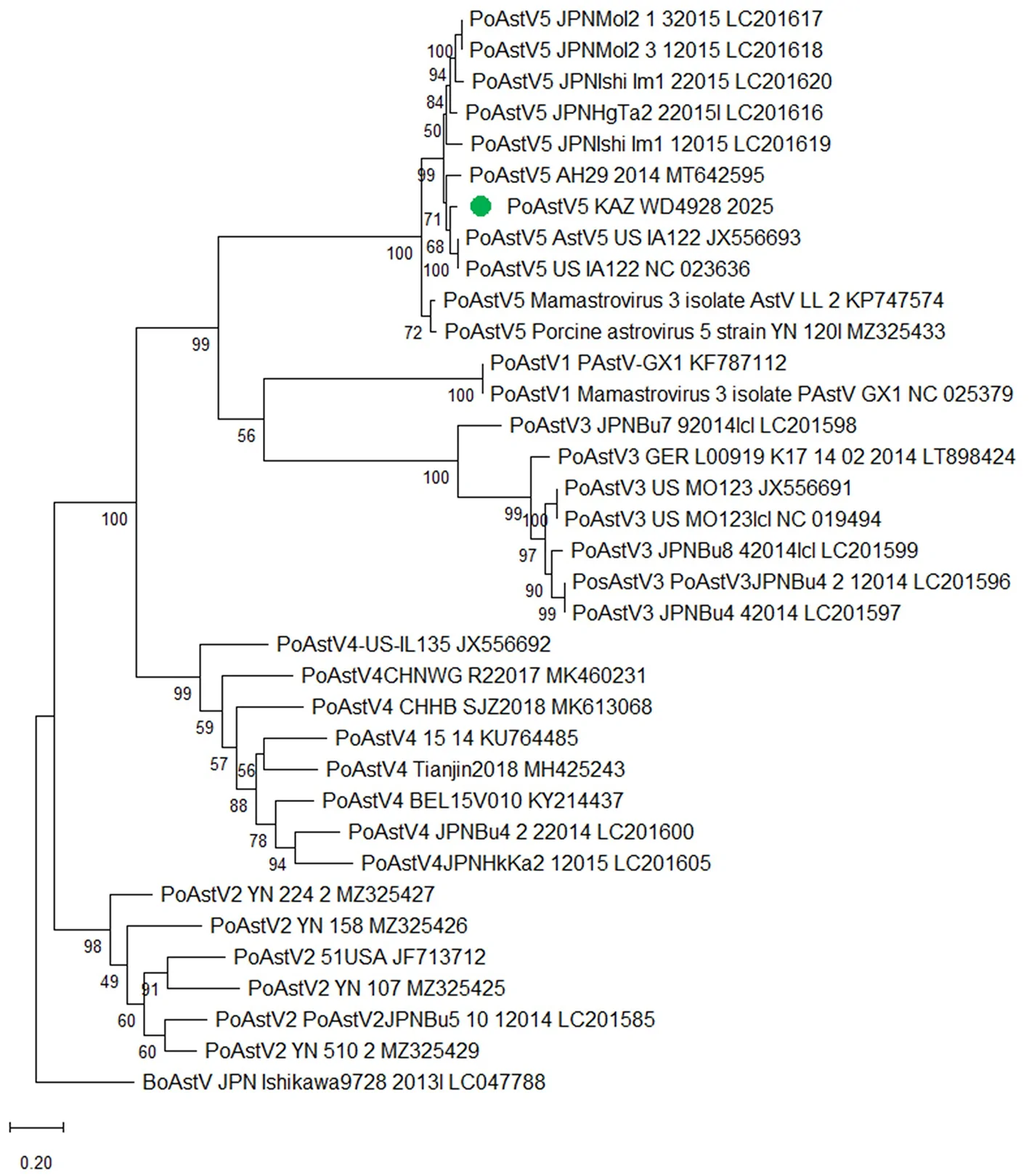
Phylogenetic analysis of Porcine astrovirus 5. The Kazakhstan isolate PoAstV5/KAZ/WD4928/2025 is indicated by a green circle on the phylogenetic tree. The Bovine astrovirus (BoAstV) sequence was used as the outgroup, enabling correct rooting of the phylogenetic tree.

The closest phylogenetic relationship for PoAstV5/KAZ/WD4928/2025 was observed with the PoAstV5 AH29 (MT642595) and PoAstV5 US IA122 (JX556693; NC_023636) strains. The node linking the Kazakhstan isolate to these strains had a bootstrap support of 68%, whereas the affiliation of the studied isolate with the PoAstV-5 clade was supported by higher bootstrap values at the upstream nodes (71–99%) (Figure 4). The phylogenetic tree showed clear separation of sequences into PoAstV-1, PoAstV-2, PoAstV-3, PoAstV-4, and PoAstV-5 species, consistent with the current classification of porcine astroviruses.

**Figure 5:**
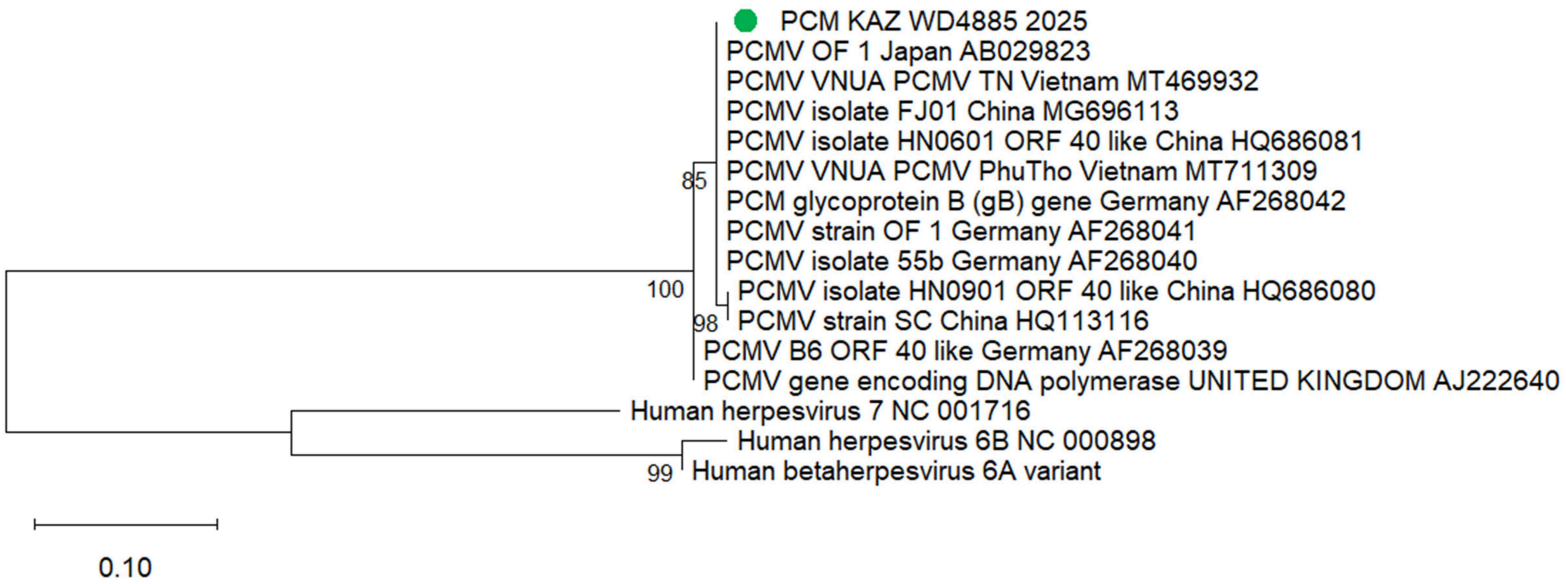
Phylogenetic analysis of Porcine Cytomegalovirus. Phylogenetic tree constructed based on the nucleotide sequences of the Porcine cytomegalovirus isolate KAZ WD4885 2025 and reference PCMV strains. The scale bar represents the number of nucleotide substitutions per site. The green circle indicates the isolate obtained in this study.Human herpesvirus 6A, 6B, and 7 sequences were used as the outgroup.

The PCMV isolate KAZ WD4885 2025 confidently clusters within the Porcine cytomegalovirus (PCMV) species, forming a sister pair with the Chinese PCMV isolate BJ09 (NC 022233) with 100% bootstrap support, which unequivocally confirms its species assignment (Figure 5). All PCMV sequences form a single monophyletic cluster (99% support), revealing pronounced geographic structure: isolates from Germany (AF268039–AF268042) and the USA (B6, AF268039) form a separate subclade with 75–99% support, whereas Chinese isolates (HQ686080, HQ686081, HQ113116, NC 022233) are distributed across several independent branches.

**Figure 6:**
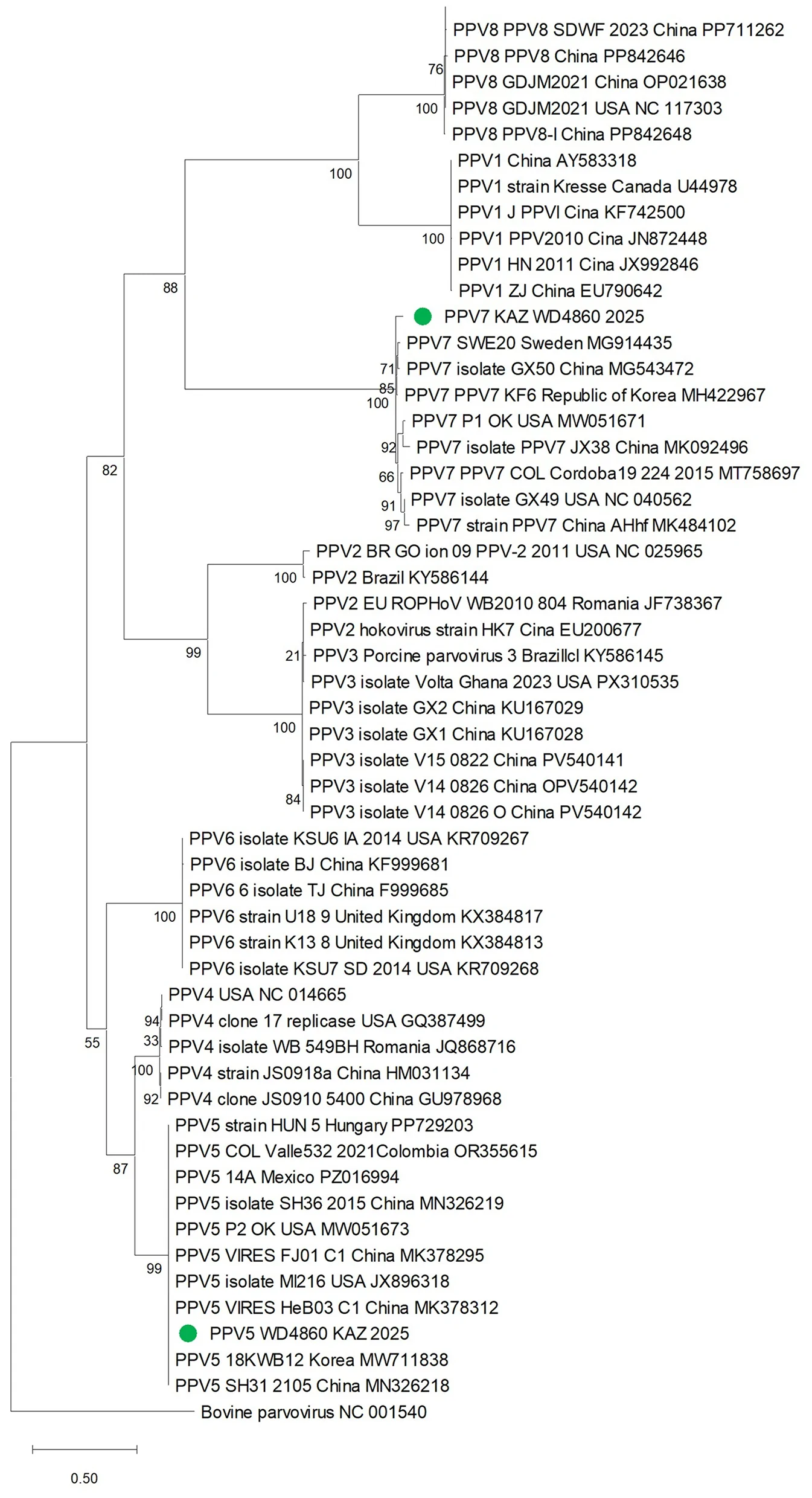
Phylogenetic tree of porcine parvoviruses (PPV1–PPV8) constructed using the maximum likelihood method based on nucleotide sequences. Bovine parvovirus (NC_001540) was used as the outgroup. Sequences obtained in this study are marked with green markers.

Phylogenetic analysis revealed a clear division of the studied sequences into clades corresponding to the PPV1–PPV8 species (Figure 6). The PPV7 WD4860/KAZ/2025 sequence obtained in this study was included in the PPV7 clade and formed a cluster with previously described isolates from China, Sweden, the Republic of Korea, the United States, and Argentina. This confirms that the identified Kazakhstan isolate belongs to the PPV7 species and indicates its genetic relationship to currently circulating variants of this virus. The PPV5 WD4860/KAZ/2025 sequence clustered within the PPV5 clade along with isolates from China, the Republic of Korea, the United States, Mexico, Hungary, and Colombia.

**Figure 7:**
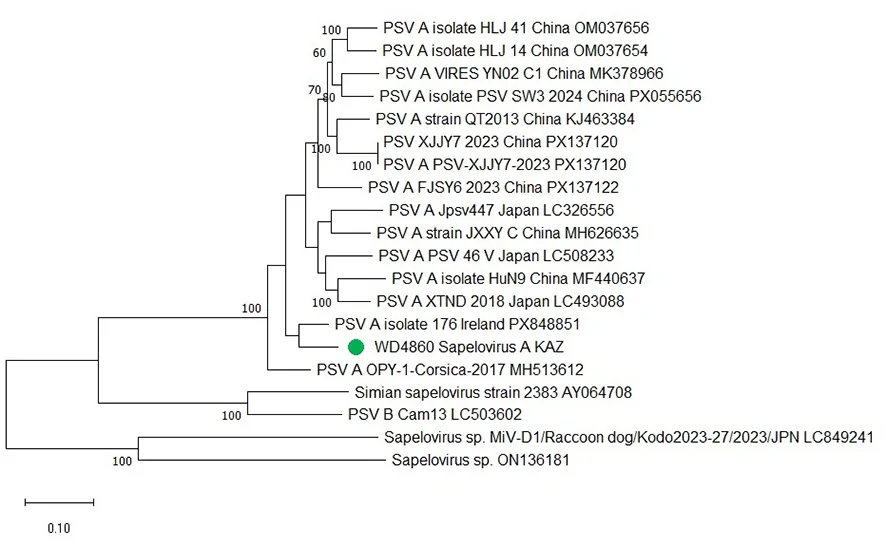
Phylogenetic tree of Porcine sapelovirus A (PSV-A) strains constructed using the maximum likelihood method based on the complete coding sequences of the genome. The Kazakhstan PSV isolate KAZ WD4860/2025 is marked with a green marker.

To determine the phylogenetic position of the Kazakhstan PSV isolate KAZ WD4860/2025, an analysis of the complete coding sequences of Porcine sapelovirus was conducted. As a result of the analysis, all PSV-A strains studied were divided into two main phylogenetic groups. The first group included predominantly isolates from China and Japan, forming a separate cluster with moderate to high statistical support (bootstrap 60–100%). The Kazakhstan PSV strain KAZ WD4860/2025 formed a cluster with the Irish PSV isolate (Figure 7). These results indicate that the Kazakhstan isolate is more closely related to European strains than to strains circulating in East Asia.

**Figure 8:**
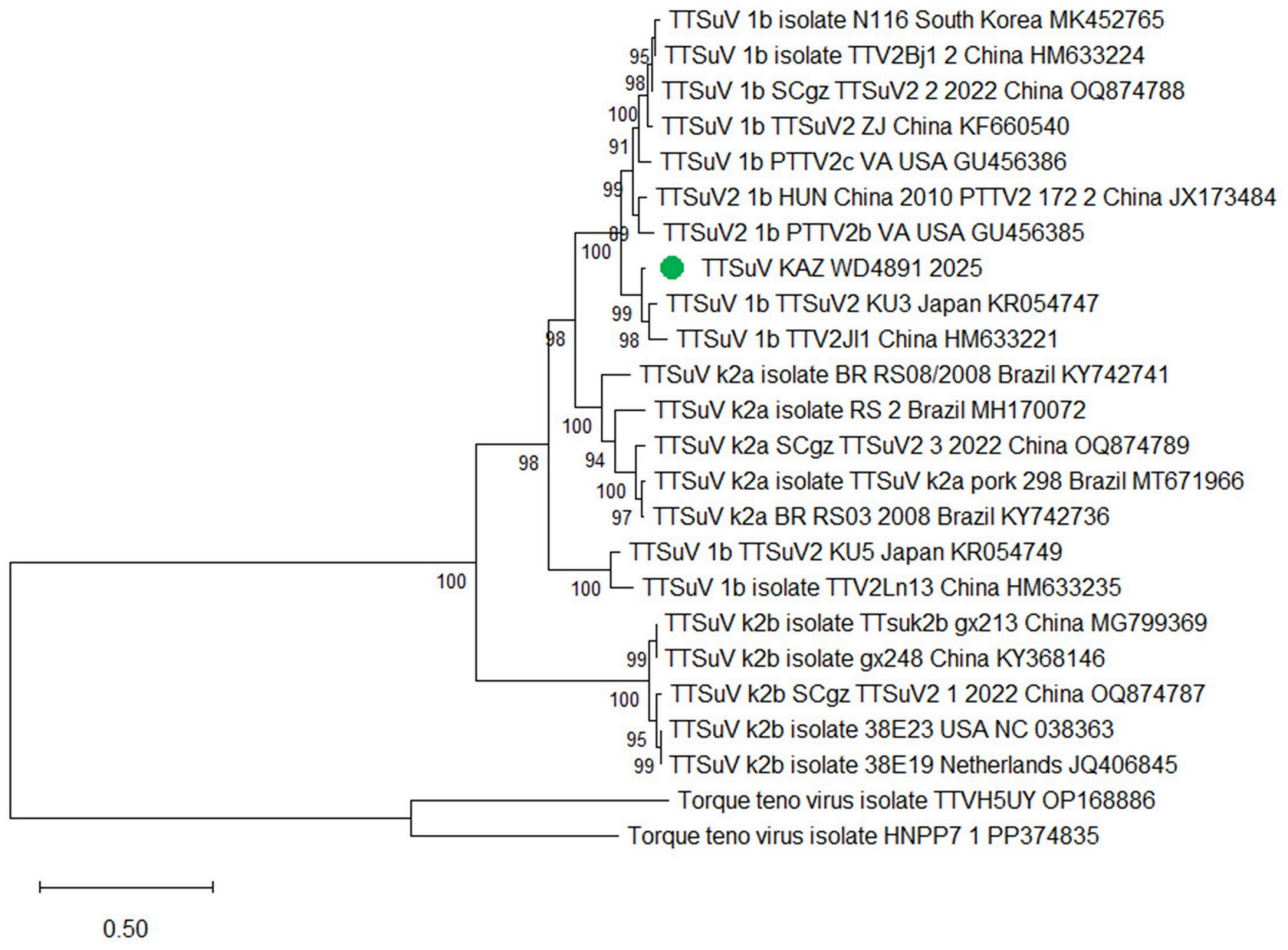
Phylogenetic tree constructed using the Maximum Likelihood method based on TTSuV whole-genome sequences. The Kazakhstan isolate TTSuV KAZ (WD4891/2025) is highlighted in green. Human Torque tenovirus sequences were used as the outgroup.

The Kazakhstan isolate TTSuV KAZ (WD4891/2025) was assigned to the TTSuV1b genotype and clustered with previously characterized strains from China, Japan, South Korea, and the United States Figure 8). The closest phylogenetic relationship was observed with the Japanese isolate KU3 (KR054747) and the Chinese isolate TTV2J11 (HM633321). The node connecting these sequences had high bootstrap support (99%), indicating a close evolutionary origin for these viruses. No significant geographic clustering was observed within the TTSuV1b genotype, as isolates from different regions of the world formed common phylogenetic groups.

## Discussion

This study presents the first comprehensive metagenomic characterization of the porcine respiratory virome on industrial farms in northern Kazakhstan, based on high-throughput sequencing of 150 nasopharyngeal swabs. Viral sequences were identified from five families, *Parvoviridae, Herpesviridae, Picornaviridae, Astroviridae*, and *Anelloviridae*, confirming the high viral diversity of the porcine upper respiratory tract and the co-circulation of DNA and RNA viruses in swine populations under intensive farming conditions.

Worldwide studies have shown that high piglet mortality during acute and chronic viral diarrhea is associated with astroviruses, bocaviruses, caliciviruses, kobuviruses, and sapeloviruses (30–32). Metagenomic sequencing is a highly efficient method for identifying known and novel viruses. Analysis of 4,650 metagenomic samples from the porcine enteric virome yielded a database of approximately 5.5 million viral sequences across approximately 48,000 viral taxa (33, 34).

The *Astroviridae* family includes the genera *Avastrovirus* and *Mamastrovirus*, which differ in host range (35). A meta-analysis reports a global prevalence of porcine astrovirus (PAstV) of 28.19%, with marked regional variation and a high rate of coinfections. PAstV also circulates widely in both healthy and sick animals, raising questions about its direct diagnostic value (36). Modern NGS studies confirm its high genetic variability and global distribution (37–39). At least five PAstV species have been described (40), and some variants have been found not only in the intestine but also in the respiratory tract (41, 42). These findings support the possibility of systemic spread and involvement in coinfection. PAstV is considered a potential zoonotic agent because astroviruses in general can cross species boundaries and show phylogenetic relationships with human strains (43–46).

Viruses in the *Picornaviridae* family are also widespread among pigs, although their pathogenicity remains poorly understood (47). Members of this family (Porcine teschovirus, sapelovirus, and enterovirus G) circulate widely in both clinically healthy and sick animals and often co-infect (48–50). Porcine teschoviruses are associated with varying degrees of central nervous system damage in pigs, causing porcine polioencephalomyelitis. The high neurotropism of these viruses and their ability to cause severe neurological disorders are supported by numerous reports of outbreaks with clinical signs of ataxia, paresis, and paralysis (51–53). Sapeloviruses (Sapelovirus A) are also considered potentially neurotropic; however, their role in central nervous system pathology remains less certain. Reported cases of CNS damage, including encephalomyelitis and neurological symptoms, are much less common and likely depend on viral strain characteristics and host factors (54–57).

Given that sapeloviruses and astroviruses are primarily enteric pathogens transmitted via the fecal-oral route, their detection in nasopharyngeal swabs in this research may partly reflect transient oro-nasal contamination arising from natural swine behaviors such as rooting, coprophagy, and close pen contact, rather than genuine respiratory tract tropism or infection. This ecological consideration should be taken into account when interpreting these viruses as components of the respiratory virome rather than as evidence of systemic or respiratory-tract replication.

The detection of *Parvoviridae* sequences in nasopharyngeal samples, including Porcine parvovirus 5 and 7 and Porcine bocavirus (PBoV), highlights the diversity of viral nucleic acids within the porcine viral ecosystem. At least eight parvovirus species (PPV1–PPV8) have been found to circulate in pigs, suggesting significant evolutionary diversification of this viral group (58). Of particular interest is PPV7, which has been reported as widely prevalent and frequently coinfected with circoviruses (PCV2/PCV3). A possible association between PPV7 and the circovirus-associated disease complex (PCVAD) was proposed, although this study's cross-sectional design in clinically unassessed animals does not permit inference about disease association in the present isolates. Likewise, reports of PPV7 detection in reproductive tissues and semen have raised the hypothesis of a role in reproductive disorders, but this remains an external literature observation rather than a finding of the current dataset (59, 60).

To place the Kazakhstan PPV5 and PPV7 findings within a broader Eurasian epidemiological context, comparison with recent Russian data is informative. Anoyatbekova et al. (61) reported the first molecular detection of PPV5 in domestic pigs in Russia, screening 984 serum samples from 20 farms across 10 regions and finding an overall detection rate of 8.9%. Phylogenetic analysis revealed high nucleotide identity between Russian isolates and strains from the USA, Colombia, and China. Similarly, Komina et al. (62) characterized porcine parvoviruses (PPV1–7) in the Russian wild boar population, identifying PPV7 as the most abundant species (59.3% of animals) alongside PPV3 (49.1%). They reported that Russian PPV5 sequences did not cluster with any local clade, instead grouping with international strains, a pattern that parallels the lack of geographically distinct clustering observed for the Kazakhstan PPV5 and PPV7 isolates in the present study. Together, these findings from a neighboring and epidemiologically connected region reinforce the interpretation that PPV5 and PPV7 circulate as globally distributed, genetically homogeneous lineages across Central Asia and Eurasia rather than as regionally isolated variants, consistent with cross-border swine trade and shared genetic stock as likely drivers of viral dispersal in the region.

The simultaneous detection of PPV5, PPV7, and PSV-A sequences in a single sample underscores the complexity of viral nucleic acid co-detection in pigs. Such multiple detections may reflect simultaneous exposure to or the presence of several viral agents in the pig population, rather than confirmed concurrent active infection. These data highlight the advantage of metagenomic sequencing, which enables detection of multiple viruses in a single sample without preselecting specific targets. Porcine bocavirus has been reported elsewhere to frequently co-occur with other respiratory and enteric pathogens, including PCV2 and PRRSV, and to exhibit pronounced genetic variability and recombination (63–65); these genomic features are consistent with ongoing viral evolution, though tissue-level adaptation cannot be assessed from nasopharyngeal detection data alone.

Porcine cytomegalovirus (PCMV), a member of the *Herpesviridae* family, is of particular importance in the context of biosafety and xenotransplantation. This virus has been associated with decreased graft survival and immune/coagulation complications following reactivation from latency. While these reports underscore the general importance of donor animal screening, no clinical or transplantation outcome data were generated in the present survey, and the detected PCMV sequence should be interpreted as evidence of viral presence, not of active or clinically relevant infection in the sampled herd (66, 67).

The widespread Torque teno sus virus (TTSuV), a member of the *Anelloviridae* family, is found in various porcine tissues and is frequently detected in coinfections with other viral agents. Its pathogenic role remains unclear, and some studies have hypothesized a possible cofactor role in PCVAD/PMWS severity (68, 69); however, this hypothesis cannot be evaluated with the present data, as no clinical or disease-severity information was collected for the sampled pigs.

Viral coinfections were observed in several samples, most prominently in sample WD4860, where three viruses (PPV5, PPV7, PSV-A) were detected simultaneously. Additional co-detections of PPV5 and PPV7 were recorded in samples WD4874 and WD4881. Overall, viral sequences were identified in 21 of 150 samples (14.0%), with *Parvoviridae* members showing the highest detection rates. These patterns align with metagenomic studies reporting multi-agent coinfection as a common feature of the porcine virome. Coinfections may influence replication dynamics, modulate local mucosal immune responses, and contribute to the persistence of viral agents within a herd. The metagenomic approach demonstrated here–enabling untargeted, simultaneous detection of multiple viral families—provides a decisive advantage over conventional PCR-based diagnostics, which require predetermined targets and may miss novel or co-circulating agents. The absence of urgent viruses such as swine influenza A virus, porcine reproductive and respiratory syndrome virus, and circovirus in this metagenomic study cannot be attributed with certainty to any single factor; possible explanations include low prevalence of these agents in the sampled herds at the time of collection, their absence from the specific farms surveyed, the nasopharyngeal sampling site not capturing viruses more concentrated in the lower respiratory tract, or technical detection limits, rather than solely reflecting the health status of the sampled animals, which was not formally documented in this study.

The phylogenetic analyses consistently showed that all seven viruses for which genomes were reconstructed–PPV5, PPV7, PCMV, PSV-A, PBoV, PoAstV5, and TTSuV1b–clustered within established globally circulating lineages rather than forming novel divergent branches. The Kazakhstan PPV5 and PPV7 isolates grouped with strains previously reported from East Asia, Europe, and the Americas; the PCMV sequence fell within a monophyletic PCMV group alongside Chinese and European isolates; and the PoAstV5, PSV-A, PBoV, and TTSuV1b sequences aligned with strains described in multiple countries, reflecting broad geographic spread of these species. This pattern suggests that the viruses detected in northern Kazakhstan are part of internationally connected viral pools, likely reflecting shared genetics of commercial pig populations and movement of breeding stock, rather than locally evolved, region-specific lineages. Introduction via pigs imported from neighboring countries, primarily Russia, is a plausible route that warrants further investigation.

Among the identified viral families, two have the strongest direct evidence of cross-species relevance. Pseudorabies virus (suid herpesvirus 1) has recently been documented as a zoonotic pathogen causing severe human encephalitis and visual impairment in individuals with occupational exposure to pigs or raw pork, underscoring the value of continued virome surveillance at the human-pig interface, even though PRV was not detected in this study. Porcine cytomegalovirus (PCMV), while not shown to replicate in human cells, has been associated with graft dysfunction, coagulopathy, and early rejection in preclinical pig-to-primate xenotransplantation studies; the PCMV sequence recovered here, together with its close phylogenetic relationship to East Asian isolates, reinforces the practical relevance of rigorous PCMV screening in donor pig herds intended for biomedical use. Other viral families discussed in the literature–including porcine parvoviruses, sapeloviruses, astroviruses, and anelloviruses–have been proposed elsewhere as having theoretical zoonotic or cross-species relevance based on sequence homology or biomarker use in transplant medicine (70–73), though these associations remain speculative and are not evaluated by the present descriptive, single-region dataset. Beyond zoonotic considerations, the findings have more immediate veterinary relevance: the frequent co-detection of parvoviruses, cytomegalovirus, and other agents in clinically unassessed animals highlights the value of routine virome screening for herd health monitoring and supports incorporating mNGS into biosecurity protocols for farms engaged in breeding stock trade or import, given the apparent link between viral lineages circulating in Kazakhstan and those reported in neighboring countries.

Several limitations of this study should be acknowledged. Metagenomic sequencing detects viral nucleic acids but cannot distinguish active infection from latent persistence or transient viral shedding. The absence of a virus isolation step precludes assessment of infectivity and cytopathic potential. Clinical and pathological data were unavailable, preventing direct association of detected viruses with clinical outcomes. The reference-based assembly strategy may reduce sensitivity to highly divergent or novel viruses, potentially underestimating actual viral diversity. The detection of PERV-E, an endogenous provirus integrated into the host genome, at low abundance despite nuclease treatment indicates incomplete depletion of host genomic DNA, which may have reduced sequencing depth available for other low-abundance exogenous viruses. Negative extraction controls and no-template controls were not included in this sequencing run. Finally, the cross-sectional design and the use of a single sample type (nasopharyngeal swabs) limit inferences about systemic viral circulation and tissue tropism. Future studies incorporating longitudinal sampling, additional tissue types, and functional virological characterization will be essential to clarify the clinical relevance of the virome described here.

Despite these limitations, this study provides the first baseline characterization of the porcine respiratory virome in Kazakhstan, demonstrates the feasibility and high sensitivity of mNGS for comprehensive viral surveillance in livestock, and highlights the co-circulation of phylogenetically diverse viral agents in clinically healthy pigs–a finding with direct implications for animal health management, biosecurity, and One Health surveillance programs in the region.

## Conclusion

This study provides the first metagenomic characterization of the porcine respiratory virome on industrial farms in Kazakhstan, revealing a complex viral community composed mainly of *Parvoviridae, Herpesviridae, Picornaviridae, Astroviridae*, and *Anelloviridae*, with several viruses having nearly complete genomes reconstructed and clear phylogenetic affiliation to globally circulating lineages. The detection of frequent parvoviruses and PCMV, along with sporadic astrovirus, sapelovirus, bocavirus, and TTSuV, together with documented coinfections in clinically healthy pigs, underscores that subclinical, persistent, and co-circulating viral infections are common in intensively reared herds and likely contribute to overall respiratory health, productivity, and susceptibility to other pathogens. The identification of porcine viral families with known or suspected zoonotic and xenotransplantation relevance reinforces the One Health importance of systematic virome monitoring at the human–pig interface. Together, these findings demonstrate that metagenomic next-generation sequencing is a powerful tool for epizootic surveillance and molecular monitoring of livestock viruses, and they establish a baseline dataset for future longitudinal studies aimed at linking specific virome configurations to clinical disease, economic impact, and cross-species transmission risk in Kazakhstan's pig industry.

## Data Availability

The datasets generated for this study are available in the GenBank database under accession numbers PZ600358-PZ600362.
